# Fast Segmentation of Metastatic Foci in H&E Whole-Slide Images for Breast Cancer Diagnosis

**DOI:** 10.3390/diagnostics12040990

**Published:** 2022-04-14

**Authors:** Muhammad-Adil Khalil, Yu-Ching Lee, Huang-Chun Lien, Yung-Ming Jeng, Ching-Wei Wang

**Affiliations:** 1 Graduate Institute of Applied Science and Technology, National Taiwan University of Science and Technology, Taipei 106335, Taiwan; m.adilkhalil13@gmail.com (M.-A.K.); d10522201@mail.ntust.edu.tw (Y.-C.L.); 2 Department of Pathology, National Taiwan University Hospital, Taipei 100229, Taiwan; lienhc@ha.mc.ntu.edu.tw (H.-C.L.); mrna0912@yahoo.com.tw (Y.-M.J.); 3 Graduate Institute of Biomedical Engineering, National Taiwan University of Science and Technology, Taipei 106335, Taiwan

**Keywords:** breast cancer segmentation, hierarchical deep learning framework, histopathological images, lymph node metastases, whole-slide image analysis

## Abstract

Breast cancer is the leading cause of death for women globally. In clinical practice, pathologists visually scan over enormous amounts of gigapixel microscopic tissue slide images, which is a tedious and challenging task. In breast cancer diagnosis, micro-metastases and especially isolated tumor cells are extremely difficult to detect and are easily neglected because tiny metastatic foci might be missed in visual examinations by medical doctors. However, the literature poorly explores the detection of isolated tumor cells, which could be recognized as a viable marker to determine the prognosis for T1NoMo breast cancer patients. To address these issues, we present a deep learning-based framework for efficient and robust lymph node metastasis segmentation in routinely used histopathological hematoxylin–eosin-stained (H–E) whole-slide images (WSI) in minutes, and a quantitative evaluation is conducted using 188 WSIs, containing 94 pairs of H–E-stained WSIs and immunohistochemical CK(AE1/AE3)-stained WSIs, which are used to produce a reliable and objective reference standard. The quantitative results demonstrate that the proposed method achieves 89.6% precision, 83.8% recall, 84.4% F1-score, and 74.9% mIoU, and that it performs significantly better than eight deep learning approaches, including two recently published models (v3_DCNN and Xception-65), and three variants of Deeplabv3+ with three different backbones, namely, U-Net, SegNet, and FCN, in precision, recall, F1-score, and mIoU (p<0.001). Importantly, the proposed system is shown to be capable of identifying tiny metastatic foci in challenging cases, for which there are high probabilities of misdiagnosis in visual inspection, while the baseline approaches tend to fail in detecting tiny metastatic foci. For computational time comparison, the proposed method takes 2.4 min for processing a WSI utilizing four NVIDIA Geforce GTX 1080Ti GPU cards and 9.6 min using a single NVIDIA Geforce GTX 1080Ti GPU card, and is notably faster than the baseline methods (4-times faster than U-Net and SegNet, 5-times faster than FCN, 2-times faster than the 3 different variants of Deeplabv3+, 1.4-times faster than v3_DCNN, and 41-times faster than Xception-65).

## 1. Introduction

Breast cancer is considered the leading cause of death for women globally [1], and according to the report by the American Cancer Society, 42,690 people in the United States of America are expected to die due to breast cancer in 2020 [2]. The prognosis of a breast cancer patient is determined by the extent of metastases, or the spreading of cancer to the other parts of the body from where it initially began [3]. Metastases usually happen when cancer cells split from the main tumor and enter the blood circulation system or the lymphatic system. The TNM staging criteria are commonly adopted to classify the extent of cancer [4]. In the TNM criteria, T refers to the size of the primary tumor (T-stage); N describes whether the cancer has spread to regional lymph nodes (N-stage); M describes whether the cancer has spread to different parts of the body (M-stage) [5]. Metastases can be divided into one of these three categories, including macro-metastases (size greater than 2 mm), micro-metastases (metastatic size greater than 0.2 mm, but no greater than 2.0 mm), and isolated tumor cells (ITCs, metastatic size no greater than 0.2 mm) [6].

The status of a tumor is commonly determined by examining routine histopathological H–E slides, but in many cases, additional costly immunohistochemical (IHC) staining is required to clarify unclear diagnoses of H–E slides [7]. However, the manual detection of cancer in a glass slide under a microscope is a time-consuming and challenging task [8]. At present, we are able to examine pathological images using computer-based algorithms by converting glass slides into whole-slide images (WSI). Dihge [9] predicted lymph node metastases in breast cancer by gene expression data, mixed features, and clinicopathological models to recognize patients with a low risk of metastases and thereby save them from a sentinel lymph node biopsy (SLNB). Shinden [10] proposed using y-glutamyl hydroxymethyl rhodamine green as a new fluorescent method to diagnose lymph node metastases in breast cancer and achieved a sufficiently high specificity (79%), negative predictive value (99%) and sensitivity (97%), proving it useful for cancer diagnosis. Dihge’s [9] and Shinden’s [10] methods require some additional data, such as gene expression data, mixed features, and expensive bio-markers, which make them impractical for clinical usage using economical histopathological slides to detect lymph node metastases. In this work, we propose a method that uses routine H–E slides for lymph node metastasis segmentations.

WSIs are extremely large: a glass slide scanned at 20× magnification produces images that are several gigapixels in size; around 470 WSIs contain nearly the same number of pixels as the whole ImageNet. When confronted with the huge amounts of information contained in large slides, even experienced pathologists are prone to misdetect features and make mistakes. As a result, qualified cancer diagnoses demand peer review and consensus, which can be costly to satisfy in hospitals and small cancer centers with a shortage of trained pathologists. To enhance performance and overcome these weaknesses, deep learning has been presented in various studies [11,12]. Deep learning has the advantage of creating high-level feature extraction and image recognition from raw images, and deep learning algorithms are being used to diagnose, classify, and segment cancer. For example, Yu et al. [13] used regularized machine learning methods to select the top features and to distinguish shorter-term survivors from longer-term ones. Coudray et al. [14] classified lung tissue slides into LUSC, LUAD, or normal lung tissue using Inception v3.

**Figure 1:**
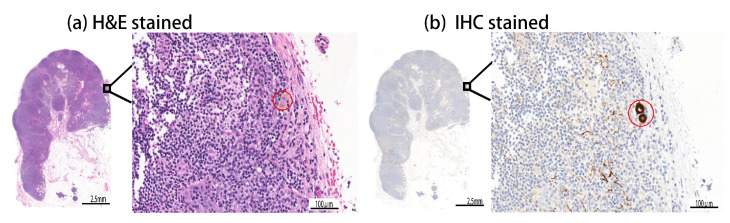
Challenges in finding tiny metastatic foci in gigapixel H–E WSIs. The segmentation of tiny micro metastases (denoted by red circles in (**a**) an H–E slide and (**b**) the associated IHC slide) is as challenging as finding a needle in a haystack. (**a**) Even in the high-magnification H–E image, ITCs are difficult to locate in humans. (**b**) In the high-magnification IHC image, metastases are visible as brown spots.

In breast cancer diagnosis, micro-metastases and ITCs are extremely difficult for medical experts to examine on H–E samples, and are highly likely to be neglected because of their tiny size, with respect to the massive dimensions of WSIs. As shown in Figure 1a, ITCs are vastly difficult for a human to find on the routinely used H–E whole-slide image. Therefore, in addition to the H–E staining, an additional expensive data-preparation and examination process, based on IHC staining for cytokeratin, is required to identify ITCs in lymph nodes, as shown in Figure 1b, where ITCs are notably visible as brown spots. The goal of this research is to develop an automated method for the fast, efficient, and accurate segmentation of breast cancer in routinely used H–E WSIs. In evaluation, the proposed method is demonstrated to be able to identify even tiny metastatic foci of challenging samples with ITCs or micro-metastases. To avoid human bias, IHC-stained slides are used to produce a reliable and zero-bias reference standard in this study, and we have collected 188 WSIs, including 94 pairs of H–E and IHC slides. The H–E slides are split into training and testing sets for training and evaluation. We compare the performance of the proposed method with eight popular or recently published deep learning methods, including SegNet [15], U-Net [16], FCN [17], and three variants of Deeplabv3+ [18] with three different backbones, which are MobileNet [19], ResNet [20], and Xception [21], as well as with two recently published models, i.e., v3_DCNN [22] and Xception-65 [23], for the segmentation of breast cancer in routinely used H–E WSIs.

The main contributions of this paper can be summarized as follows:

- We present an efficient and robust deep learning model for the segmentation of breast cancer in H–E-stained WSIs. The experimental results show that the proposed method significantly outperforms the baseline approaches for the segmentation of breast cancer in H–E-stained WSIs (p<0.001);
- Our framework is demonstrated to be capable of detecting tiny metastasis foci, such as micro-metastases and ITCs, which are extremely difficult to find by visual inspection on H–E-stained WSIs. In comparison, the baseline approaches tend to fail in detecting tiny metastasis foci;
- By leveraging the efficiency of a tile-based data structure and a modified fully convolutional neural network model, the proposed method is notably faster in gigapixel WSI analysis than the baseline approaches, taking 2.4 min to complete the whole slide analysis utilizing four NVIDIA Geforce GTX 1080Ti GPU cards and 9.6 min using a single NVIDIA Geforce GTX 1080Ti GPU card.

This paper is organized as follows. Section 2 presents the related works; Section 3 describes the details of the materials and methods used; Section 4 presents the results, including a comparison with the baseline approaches; Section 5 provides discussions and presents the significance of the work; Section 6 draws the conclusion and presents the future research directions.

## 2. Related Works

In recent years, due to sensational advancements in computer power and image-scanning techniques, more researchers evaluate their algorithms on WSI datasets. Bejnordi et al. [24] proposed a multiscale superpixel method to detect the ductal carcinoma in situ (DCIS) in WSIs. Huang et al. [25] proposed a convolutional network with multi-magnification input images to automatically detect hepatocellular carcinoma (HCC). Celik et al. [26] used pre-trained deep learning models, ResNet-50, and DenseNET-161 for the automated detection of invasive ductal carcinoma detection. Gecer et al. [27] proposed deep convolutional networks (DCNN) for the detection and classification of breast cancer in WSIs. Firstly, they used a saliency detector that performs multi-scale localization of relevant ROI in a WSI. After that, a convolutional network classifies image patches into five diagnostic categories (atypical ductal hyperplasia, ductal carcinoma in situ, non-proliferative or proliferative changes, and invasive carcinoma). In the end, slide-level categorization and pixel-wise labeling are performed by fusing classification and saliency maps. Lin et al. [28] proposed a framework for the fast and dense scanning of metastatic breast cancer detection in WSI. However, Lin’s [28] method does not deal with ITCs, which are extremely difficult for medical experts to examine on H–E samples in breast cancer diagnosis, and are highly likely to be neglected because of their tiny size, with respect to the massive dimensions of the WSIs.

In 2016 and 2017, the Camelyon16 [29] and Camelyon17 challenges [3,29,30] were held, aiming to evaluate new and existing algorithms for the automated detection and classification of metastases in H–E-stained WSIs of lymph node sections. In Camelyon16, 400 WSIs and the associated annotations were provided, where 270 slides were for training and 130 slides for testing; in Camelyon17, 1399 WSIs and the associated annotations were provided, where 899 slides were for training and 500 slides for testing. Wang [31] implemented an ensemble of two GoogLeNets for patch-based metastasis detection and won Camelyon16. Firstly, they divided WSIs into patches with 256×256 pixels and then trained an ensemble of two GoogLeNet classification models to detect cancer regions.

For the fast and precise pixel-based segmentation of breast cancer, Guo et al. [22] introduced a v3_DCNN framework in 2019, which combines an Inception-v3 classification model for the selection of tumor regions and a DCNN segmentation model for refined segmentation. Guo [22] used three different patch sizes to train models, including 321×321, 768×768, and 1280×1280. They named the three DCNN models after the varied sizes of training patches: DCNN-321, DCNN-768, and DCNN-1280, respectively. Priego et al. [23] proposed a patch-based deep convolutional neural network (DCNN), together with an encoder-decoder with a separable atrous convolution architecture for the segmentation of breast cancer in H–E-stained WSIs.

## 3. Materials and Methods

In this section, we describe the datasets and the proposed deep learning-based framework with a modified fully convolutional network, which is trained using transfer learning, boosting learning, boosted data augmentation, and focus sampling techniques to boost its performance for the segmentation of breast cancer on H–E-stained WSIs. This section is divided into five subsections: in Section 3.1, the datasets are described; in Section 3.2, the transfer learning, boosting learning, boosted data augmentation, and focus sampling techniques of the proposed method are described; in Section 3.3, the proposed deep learning-based framework is described; in Section 3.4, the modified fully convolutional network is described; in Section 3.5, the implementation details of the modified fully convolutional network and the baseline approaches are described.

### 3.1. The Dataset

**Figure 2:**
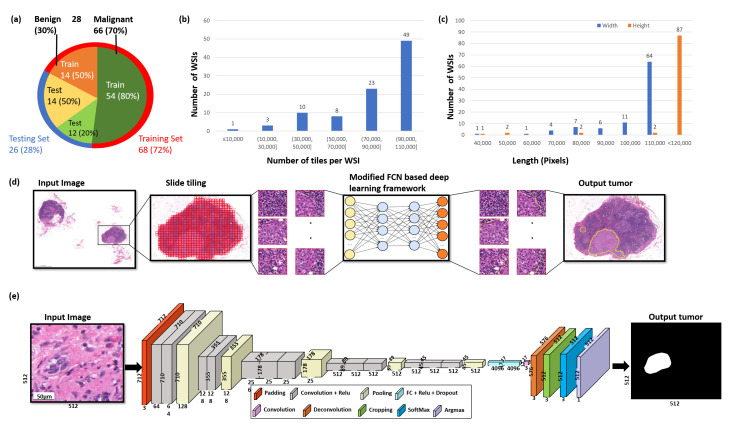
The overview of the data and the proposed deep learning framework presented in this study. (**a**) Distribution of data between malignant and benign samples and for training and testing data, respectively. (**b**) The number of tiles delivered per WSI. (**c**) The width and height distributions of the WSIs are shown in blue and orange, respectively. (**d**) The proposed deep learning framework for the segmentation of breast cancer. Firstly, Otsu’s method is used to threshold the slide image to efficiently discard all background noise. Secondly, each WSI is formatted into a tile-based data structure. Thirdly, the tiles are then analyzed by a deep convolutional neural network to produce the breast cancer metastasis segmentation results. (**e**) Illustration of the proposed modified FCN architecture.

The dataset is directly obtained from the National Taiwan University Hospital with an ethical approval (NTUH-REC 201810082RINB) by the research ethics committee B of the National Taiwan University Hospital on 8 March 2019, containing 188 H–E- and IHC CK(AE1/AE3)-stained lymph slides. Out of 188 WSIs, 94 slides are H–E-stained and the other 94 are IHC CK(AE1/AE3)-stained WSIs. The dimensions of the slides are, on average, 113,501×228,816 pixels, with a physical size of 25.11×50.63 mm${}^{2}$. All breast cancer tissue slides with lymphatic metastases were scanned using a 3DHISTECH Pannoramic (3DHISTECH Kft., Budapest, Hungary) scanner at ×20 objective magnification. All the annotations were made by two expert pathologists with the guidance of IHC biomarkers. The whole dataset was split into 2 separate subsets for training and testing, including 68 slides for the training set (≈72%), from which 54 are malignant slides and 14 are benign slides, and 26 slides for the testing set (≈28%), from which 12 are malignant slides and 14 are benign slides (Figure 2a), which ensures the models are never trained and tested on the same sample. For training the AI models, around 0.02% of the malignant tissue samples and 0.01% of the benign tissue samples were used in the training set. Detailed information about the distribution of the WSIs is shown in Figure 2b,c. For a quantitative evaluation, the IHC slides were used to produce a reliable reference standard.

### 3.2. Transfer Learning, Boosting Learning, Boosted Data Augmentation, and Focusing Sampling

#### 3.2.1. Transfer Learning

**Figure 3:**
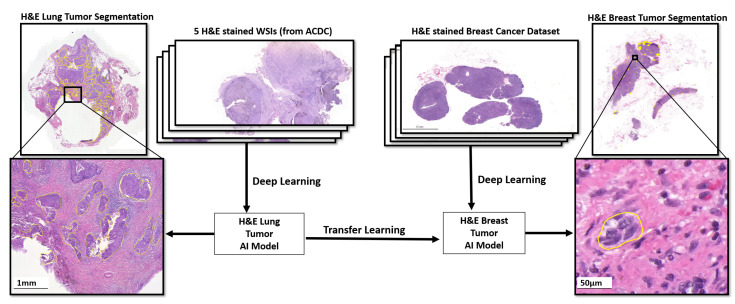
The H–E-stained breast cancer segmentation model is trained through transfer learning based on a lung tumor AI model which learned from five H–E WSIs of the IEEE ACDC challenge dataset. The pre-trained weights of the H–E lung tumor AI model are used as initial weights to train the proposed EBUS-TBNA lung tumor AI model for the segmentation of the H–E-stained breast dataset.

Transfer learning is a machine learning technique in which a model trained on one task is repurposed to the second related task by adding some modifications [32]. For instance, one can visualize using an image segmentation model trained on ImageNet, which contains thousands of classes of different objects, to begin task-specific learning for cancer detection. Transfer learning is usually useful for tasks in which enough training samples are not available to train a model from scratch, such as medical image segmentation for rare or emerging diseases [32]. For models based on deep neural networks, this is, particularly, the situation that requires a large number of parameters for training. By utilizing transfer learning, the model parameters start with already-good initial weights that only need some small alterations to be better curated towards the second task. The transfer learning approach has been frequently used in pathology, for example, in mitochondria segmentation [33], organelle segmentation [34], and breast cancer classification [35]. In this study, we use the pre-trained weights of the lung cancer segmentation model trained using five randomly selected WSIs from an H–E-stained lung dataset provided by the Automatic Cancer Detection and Classification (ACDC) in the Whole-Slide Lung Histopathology challenge, held with the IEEE International Symposium in Biomedical Image (ISBI) in 2019 [36], as initial weights to train the proposed model for the segmentation of tumors in an H–E-stained breast dataset, as shown in Figure 3. We assumed that this pre-trained network could be able to recognize tumor tissue morphology. As a result, this pre-trained model serves as the backbone architecture for transfering information in order to find breast tumor tissues. However, to train a model with such a small dataset, the following boosting learning strategy is designed.

#### 3.2.2. Boosting Learning

Given a training set $T:\{\left(a_{d},b_{d}\right)\}$, where $a_{d}$ represents the instance data and $b_{d}\in B:\left\{0,1\right\}$ represents the label, a learner η, and the base deep learning model *C*, the proposed boosting learning produces the final AI model $\theta^{*}\left(a\right)$ by the following steps. Firstly, create a new set $T_{1}:\left\{\left(u_{e},l_{e}^{1}\right)\right\}$ with instance weight $l_{e}$, where $u_{e}:{\left\{a_{r}\right\}}_{r=1,\ldots ,U\times U}$ represents a tile; U=512. Each instance weight $l_{e}^{1}$ is initialized with an IoU-based attention weighting function ϑ for further training.

(1) $$l_{e}^{1}=\left\{\begin{array}{cc} 1 & ,\vartheta \geq \gamma \\ 0 & ,otherwise \end{array}\right.$$

where $\vartheta =\frac{\sum_{a_{d}\in b_{d}}f_{i}}{\mathrm{card}(u_{e})}$; γ=0.05.

Then, iteratively for h=1,…,H, build a base model $\theta_{h}=\eta \left(T_{h}\right)$. The sample weights $\left\{l_{e}^{h+1}\right\}$ are continuously modified and formulated by increasing the attention weights of false positives and false negatives of $\theta_{h}$.

(2) $$l_{e}^{h+1}=\left\{\begin{array}{cc} l_{e}^{h}+\chi & ,\frac{\sum_{a_{r}\in u_{e}}{1|}_{\theta_{h}\left(a_{r}\right)\neq f_{r}}}{U\times U}\geq \gamma \\ l_{e}^{h} & ,otherwise \end{array}\right.$$

#### 3.2.3. Boosted Data Augmentation

Next, we devised a boosted data augmentation based on the sample attention weights $\left\{l_{e}^{h+1}\right\}$ and produced new data $T_{h+1}$. Data augmentation was applied to enlarge the training set with additional synthetically modified data by manipulating the rotation per 5°, and 5 times with an increment of 90°, mirror-flipping it along the horizontal and vertical axes, and adjusting the contrast (random contrast, range 0% ± 20%), the saturation (random saturation, range 0% ± 20%), and the brightness (random brightness, range 0% ± 12.5%).

#### 3.2.4. Focusing Sampling

When the training data is partially labeled, causing many unlabeled tissues of interest to be wrongly defined as background or content of no interest, this severely confuses AI learners during supervised learning and deteriorates the performance of the output AI models. To deal with this issue, we have added an IoU-based focusing sampling mechanism for computing the gradients effectively. A number of unlabeled cells will now not be used as negative samples for training to confuse learning, but are arranged as ignored samples. This will not only help the learning be more focused, but also speed up the learning time. Moreover, we increase the learning efforts for false positive and false negative predictions, and further, add variations of the FPs and FNs to assist the AI to learn better, deal with its weakness, and produce improved AI models.

### 3.3. Whole-Slide Image Processing

Figure 2d shows the deep learning-based approach for the segmentation of metastatic breast cancer from WSIs. Firstly, Otsu’s approach will be utilized to filter the WSI to exclude all background noise, thereby substantially lowering the amount of processing per slide. Then, each WSI is formulated as a tile-based data structure $U=\left\{u_{i,j}\right\}$ to deal with gigapixel data efficiently, where *u* represents a tile unit; *i* and *j* represent the row and column number of a tile, respectively.

Next, a deep learning model C is built using a modified fully convolutional neural network for fast WSI analysis, which is described in detail in the next section. The tiles are processed by the proposed deep convolutional neural network *C* to obtain the probabilities for cancer cells, as shown in Equation (3). Then, the pixel-based segmentation result of tumor cells $O=\{o_{i,j}\left(x,y\right)\}$ is produced based on the tumor cells’ probabilities $p_{i,j}\left(x,y\right)$.

(3) $$p_{i,j}\left(x,y\right)=C\left(u_{i,j}\left(x,y\right)\right)$$

(4) $$o_{i,j}\left(x,y\right)=\left\{\begin{array}{cc} u_{i,j}\left(x,y\right) & ,\;C(u_{i,j}\left(x,y\right))=\alpha \\ \phi & ,otherwise \end{array}\right.$$

The tile size and alpha are input parameters and are empirically set as 512 × 512 and 0.5, respectively.

### 3.4. The Proposed Modified Fully Convolutional Network

**Figure 4:**
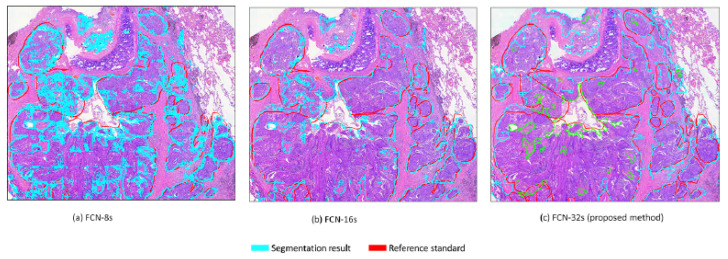
Three upsampling layers are compared. The findings of (**a**) FCN-8s and (**b**) FCN-16s are excessively fragmented when evaluated against the results of the (**c**) FCN-32s, which are the closest to the reference standard.

Fully convolutional networks (FCNs) are widely used in the field of pathology, including for counting cells in different kinds of neuropathology [37] and microscopy images [38], and for the segmentation of nuclei in histopathology images [39]. Our FCN architecture is modified based on the original FCN framework of Shelhamer [17], with two improvements. Firstly, we address the problem of insufficient GPU memory during training by using the shallow network of five layers rather than seven layers in the conventional FCN network [17]. Secondly, segmentation is required to produce a prediction for every pixel and to perform upsampling to restore the original size. Therefore, we utilize single-stream 32s upsampling to avoid excessively fragmented segmentation results, as shown in Figure 4, and to reduce the computational time for training and inference. In this work, we devise a modified FCN by utilizing the single-stream 32s upsampling as the base deep learning model to decrease GPU memory consumption, improve segmentation results, and decrease the computational time for training and inference. The modified FCN architecture has a padding layer, five convolutional blocks, a rectified linear unit (ReLU) activation function after every convolutional layer, five max-pooling layers, a deconvolutional layer, a softmax layer, and two dropout layers. The modified FCN begins with a padding layer that is used to increase the input size from 512 × 512× 3 to 712 × 712 × 3. Following the padding layer, there are five convolutional blocks that are applied in a sequential manner. The first two convolutional blocks consist of two convolutional layers with a filter size of 3 × 3 and a stride size of 1, and the remaining three convolutional blocks consist of three convolutional layers with a filter size of 3 × 3 and a stride size of 1. Each convolution layer in the convolutional block is followed by the ReLU layer. Each convolutional block is followed by a maxpooling layer with a filter size of 2 × 2 and stride size of 2. After the convolutional blocks and maxpooling layer, a deconvolutional layer with a filter size of 64 × 64 and stride size of 32 is applied to obtain the upsampled feature maps. Cropping is performed after the deconvolution layer to restore the feature maps to the same size as an input image. After cropping, softmax is used to obtain the class probabilities. At the end, an argmax function is applied on the class probabilities to produce the pixel-based class map. The detailed architecture of the modified FCN is shown in Figure 2e.

### 3.5. Implementation Details

To train the proposed technique, the model is initialized using the VGG16 model and optimized with stochastic gradient descent (SGD) optimization, and the cross-entropy function is used as a loss function. Furthermore, the proposed method is trained with the following settings: a learning rate of $1\times 10^{-10}$, dropout ratio of 0.5, and weight decay of 0.0005, respectively. The baseline approaches, including U-Net [16], SegNet [15], and FCN [17], are implemented using the Keras implementation of image segmentation models by Gupta et al. [40], initialized using a pre-trained VGG16 model, and optimized with Adadelta optimization, with the cross entropy function as a loss function. In addition, U-Net, SegNet, and FCN are trained with the following settings: a learning rate of 0.0001, dropout ratio of 0.2, and weight decay of 0.0002, respectively. For the baseline approaches, including DeepLabv3+ [18] with three different backbones, which are MobileNet [19], ResNet [20], and Xception [21], the networks are optimized using SGD optimization, with the cross-entropy function as a loss function. Furthermore, DeepLabv3+ with three backbones is trained with the following settings: a learning rate of 0.007, dropout ratio of 0.2, and weight decay of 0.00005, respectively.

## 4. Results

In this section, the evaluation metrics and the quantitative evaluation results with statistical analysis are described. This section is divided into three subsections: in Section 4.1, we describe the evaluation metrics; in Section 4.2, we present the quantitative evaluation results of the proposed method and compare the performance with the baseline approaches; in Section 4.3, we present the hardware specifications and run-time analysis of the proposed method, and compare the performance with the baseline approaches.

### 4.1. Evaluation Metrics

Four criteria are adopted to produce the quantitative evaluation of the segmentation performance, i.e., mean intersection over union (mIoU), F1-score, precision, and recall.

mIoU refers to the mean of IoUs computed on normal and tumor slides. The IoU can be formulated according to Equation (5):

(5) $$IoU=\frac{TP}{TP+FN+FP},$$

where *TP* represents the true positive, *TN* is the true negative, *FP* denotes false positive, and *FN* is the false negative.

mIoU is the mean value of IoU over all the classes in the dataset.

(6) $$mIoU=\frac{1}{q+1}\sum_{b=0}^{q}IoU_{b},$$

where *q* + 1 is the total number of classes and $IoU_{b}$ is the intersection over union of class *b*.

Precision, recall, and F1-score are computed as follows:

(7) $$Precision=\frac{TP}{TP+FP}$$

(8) $$Recall=\frac{TP}{TP+FN}$$

(9) $$F1\text{-}score=\frac{2TP}{2TP+FP+FN}$$

### 4.2. Quantitative Evaluation with Statistical Analysis

**Figure 5:**
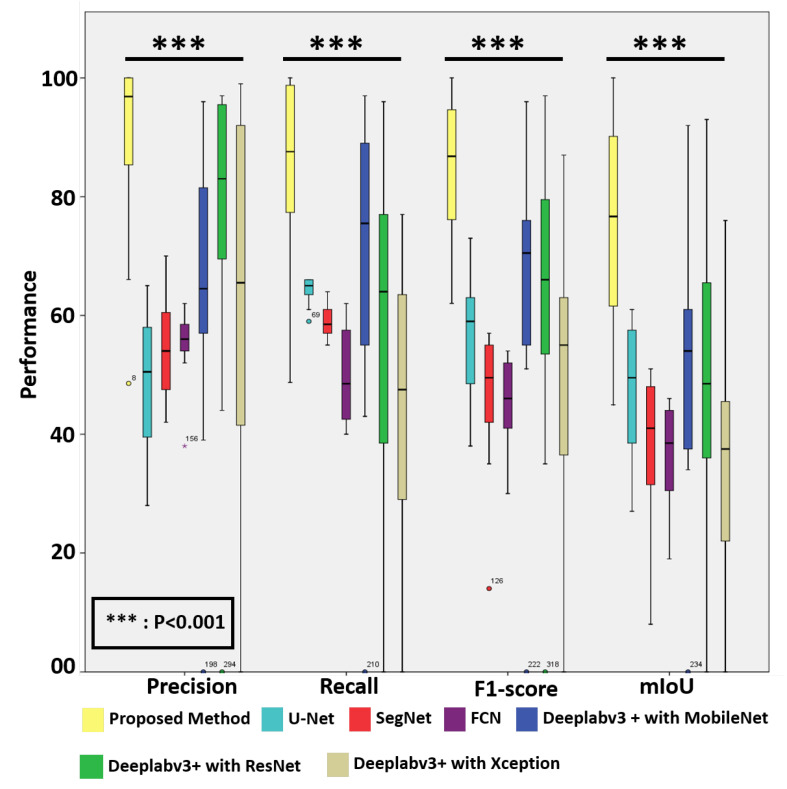
The box plot of the quantitative evaluation results in metastasis segmentation, where the outliers >1.5× the interquartile range are marked with a dot. The results of the LSD tests (p<0.001) show that the proposed method significantly outperforms the baseline approaches.

**Figure 6:**
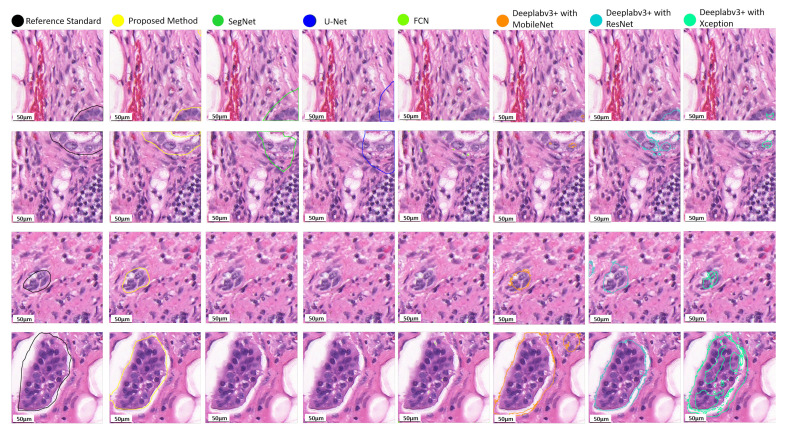
Qualitative evaluation of the metastasis segmentation results by the proposed method and the baseline approaches for breast cancer segmentation in histopathological images.

**Figure 7:**
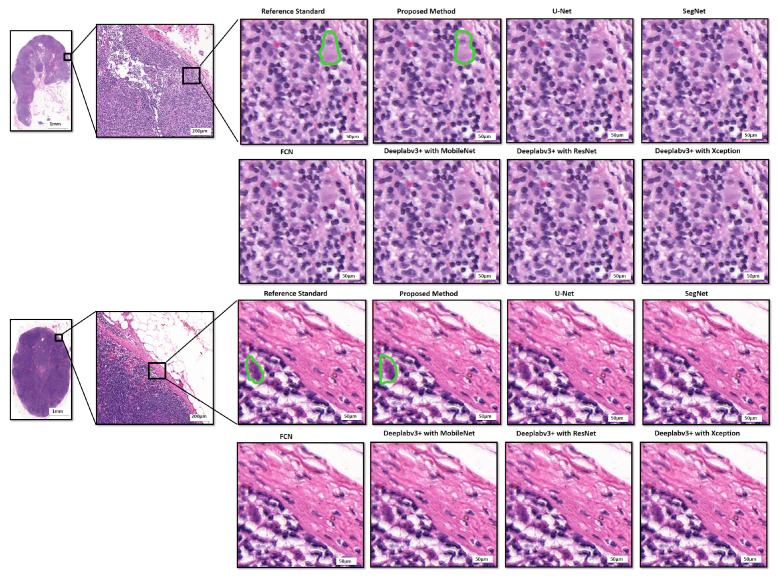
Comparison of the proposed method and the baseline approaches for the segmentation of tiny metastatic foci in challenging cases with isolated tumor cells. The results show that the proposed method is capable of effectively segmenting the tiny metastasis foci, while the baseline approaches tend to fail in segmenting the tiny metastasis foci.

For quantitative assessment, we compared the effectiveness and efficacy of the suggested approach to eight deep learning models, including two recently published models, i.e., v3_DCNN [22] and Xception-65 [23], and U-Net [16], SegNet [15], FCN [17], and three variants of Deeplabv3+ [18] with three different backbones, including MobileNet [19], ResNet [20], and Xception [21], for breast cancer segmentation in routinely used histopathological H–E WSIs, as presented in Table 1. As can be observed, the proposed approach surpasses the baseline techniques in the segmentation of breast cancer in histopathological images with 83.8% recall, 89.6% precision, 84.4% F1-score, and 74.9% mIoU, respectively. In addition, the box plots of the quantitative assessment results for breast cancer segmentation are shown in Figure 5, demonstrating that the suggested technique consistently outperforms the baseline approaches. To further demonstrate the efficacy and efficiency of the proposed method, using SPSS software, we examined the quantitative scores that were evaluated with Fisher’s least significant difference (LSD) procedure (Table 2). Based on the LSD test, the suggested approach substantially exceeds the baseline approaches in terms of precision, recall, F1-score, and mIoU (p<0.001). Figure 6 presents the visual comparison of the segmentation results of the proposed method and the baseline approaches for the segmentation of breast cancer in H–E slides. Here, we can observe a consistency between the typical segmentation results generated by the proposed method and the reference standard produced by expert pathologists, while the baseline approaches are unable to produce thhe full segmentation results of metastatic lesions. Figure 7 compares the suggested approach and the baseline approaches for the segmentation of tiny metastatic foci such as micro-metastases and ITCs in challenging cases with isolated tumor cells, demonstrating that the suggested approach is capable of effectively segmenting the tiny metastasis foci, while the reference approaches tend to fail in detecting the tiny metastasis foci.

### 4.3. Run Time Analysis

The computation time of WSI is critical for actual clinical utilization due to the massive size of WSIs. Therefore, we analyzed the overall AI inference time for processing a WSI (Table 3). Table 3 compares the hardware and computing efficiency of the suggested approach with eight deep learning approaches, including, U-Net, SegNet, FCN, and three variants of Deeplabv3+ with three different backbones, which are MobileNet, ResNet, and Xception, as well as two recently published models (v3_DCNN, Xception-65), showing that the proposed method is notably faster than the baseline approaches. For the run-time analysis of v3_DCNN and Xception-65, we referred to the reported numbers of Guo [22] and Priego [23]. As shown in Table 3, the proposed method takes 2.4 min for a WSI analysis, utilizing four NVIDIA Geforce GTX 1080Ti GPU cards, and 9.6 min using a single NVIDIA Geforce GTX 1080Ti GPU card, while the U-Net model takes 44 min, the SegNet model takes 43 min, the FCN model takes 48 min, the Deeplabv3+ with MobileNet model takes 17.2 min, the Deeplabv3+ with ResNet model takes 18.2 min, and the Deeplabv3+ with Xception model takes 17.8 min; the patch-based Xception-65 model requires 398.2 min, estimated by multiplying the time cost of 0.23 s for a single 500×500 patch by the total number of patches of a WSI, and the best model of v3_DCNN takes 13.8 min, approximated by multiplying the time cost of $5.31\times 10^{-10}$ s for a single pixel by the total number of pixels. Having the same slide dimensions and hardware equipments, the proposed method is 4-times faster than U-Net and SegNet, 5-times faster than FCN, 2-times faster than three different varaints of DeepLabv3+, 1.4-times faster than v3_DCNN, and 41-times faster than Xception-65, even with a less-expensive GPU. Altogether, the suggested technique is proved to be able to reliably detect breast cancer in H–E data and swiftly process WSIs in 2.4 min for actual clinical usage.

## 5. Discussion and Significance of the Work

### 5.1. Discussion

The histopathological H–E analysis of tissue biopsies plays a key role in the diagnosis of cancer and in devising the treatment procedure [28]. Manual pathological diagnosis is an extremely challenging, laborious, and time-consuming task. With the increasing cancer morbidity, the population of pathologists cannot fulfill the increasing demand of diagnosis. Pathologists must undertake a comprehensive evaluation of all information on a significant number of biopsy slides every day in histopathological diagnosis. More significantly, there is a considerable risk of misdiagnosis in difficult instances such as ITCs and micro-metastases. There are strong grounds to assume that digital pathology, in conjunction with artificial intelligence for CAD diagnosis, is a solution to this problem since it helps create more accurate diagnoses, shortens examination times, and reduces both pathologists’ efforts and examination costs.

Prior to Camelyon 16, there have been few studies applying deep learning to gigapixel WSIs. The majority of the solutions used image analysis on pre-selected areas of 500 × 500 pixels that were hand-picked by experienced pathologists. The computational cost is the key hurdle in employing computational approaches to diagnose gigapixel WSIs, which is why many existing algorithms are not well-suited to clinical applications. A complete and thorough automated inspection of WSIs with high accuracy may require additional time and computer resources. In this paper, we describe a quick and efficient approach for segmenting small metastases in WSIs that not only achieves state-of-the-art performance but also overcomes the primary computational cost constraint of WSI analyses. The suggested approach can finish the whole slide analysis of a big WSI with 113,501 × 228,816 pixels in 2.4 min using four NVIDIA Geforce GTX 1080Ti GPU cards, and in 9.6 min using a single NVIDIA Geforce GTX 1080Ti GPU card. More crucially, the suggested technique has been shown to be capable of recognizing ITCs (the smallest kind of metastasis), which have a high chance of misinterpretation by professional pathologists due to their small size (see Figure 7). The results of the experiments reveal that the suggested technique achieves 83.8% recall, 89.6% precision, 84.4% F1-score, and 74.9% mIoU. Furthermore, based on the LSD test, the proposed technique outperformed state-of-the-art segmentation models such as U-Net, SegNet, FCN, and three distinct variations of Deeplabv3+, as well as two newly released models, namely, v3 DCNN and Xception-65 (p<0.001).

### 5.2. Significance of the Work

The quantitative and qualitative results show that the proposed method could be a highly valuable tool for aiding pathologists in the segmentation of WSIs of breast tissues. This information could be critical for delivering suitable and personalized targeted therapy to breast cancer patients, broadening the scope and effectiveness of precision medicine, which aspires to build a multiplex strategy with patient-specific therapy. More importantly, the proposed method is shown to be capable of detecting ITCs (the smallest kind of metastasis), which could be recognized as a viable marker to determine the prognosis for T1NoMo breast cancer patients. Furthermore, the run-time analysis results show that the proposed method also overcomes the major speed bottleneck in employing computational approaches to diagnose gigapixel WSIs.

## 6. Conclusions and Future Directions

In this paper, we present a deep learning-based system for automated breast cancer segmentation in commonly used histopathological H–E WSIs. We evaluated our proposed framework using 188 WSIs, containing 94 H–E- and 94 IHC CK(AE1/AE3)-stained WSIs, which are used to create a reliable and objective reference standard. The quantitative results demonstrate that the proposed method achieves 89.6% precision, 83.8% recall, 84.4% F1-score, and 74.9% mIoU, and significantly outperforms eight baseline approaches, including U-Net, SegNet, FCN, three variants of Deeplabv3+ with three different backbones, as well as two recently published methods (v3_DCNN, and Xception-65) for the segmentation of breast cancer in histopathological images (p<0.001). Furthermore, the results show that our proposed work is capable of identifying tiny metastatic foci that have a high probability of misdiagnosis by visual inspection, while the baseline approaches tend to fail in detecting the tiny metastatic foci for cases with micro metastases or ITCs. The run-time analysis results show that the proposed deep learning framework can effectively segment the lymph node metastases in a short processing time using a low-cost GPU. With high segmentation accuracy and less computational time, our proposed architecture will help pathologists to effectively diagnose and grade tumors by increasing the diagnosis accuracy, reducing the workload of pathologists, and speeding up the diagnosis process. In the future, we anticipate that the system will be used in clinical practice to assist pathologists with portions of their assessments that are well-suited to automatic analysis, and that the segmentation will be extended to other forms of cancer. Another aspect that could be investigated in future works is the use of weakly supervised deep learning technologies for WSI analysis, which can be optimized with a limited amount of labeled training data.

## Figures and Tables

**Table 1:** The quantitative evaluation of the proposed method and the baseline approaches in the segmentation of metastases on H–E WSIs.

	Method	Score			95% C.I. for Mean	
		Mean	Std. Deviation	Std. Error	Lower Bound	Upper Bound
	Proposed method	**0.892**	0.163	0.047	0.787	0.995
Precision	U-Net [16]	0.486	0.116	0.033	0.411	0.559
	SegNet [15]	0.548	0.091	0.026	0.489	0.605
	FCN [17]	0.552	0.062	0.018	0.512	0.590
	Deeplabv3+ [18] with MobileNet [19]	0.643	0.262	0.075	0.476	0.809
	Deeplabv3+ [18] with ResNet [20]	0.613	0.354	0.102	0.388	0.838
	Deeplabv3+ [18] with Xception [21]	0.753	0.286	0.082	0.571	0.935
	Proposed method	**0.837**	0.169	0.049	0.729	0.945
Recall	U-Net [16]	0.643	0.022	0.006	0.628	0.656
	SegNet [15]	0.588	0.028	0.008	0.570	0.606
	FCN [17]	0.500	0.082	0.023	0.448	0.551
	Deeplabv3+ [18] with MobileNet [19]	0.682	0.277	0.080	0.506	0.858
	Deeplabv3+ [18] with ResNet [20]	0.440	0.261	0.075	0.274	0.606
	Deeplabv3+ [18] with Xception [21]	0.584	0.290	0.083	0.399	0.768
	Proposed method	**0.844**	0.127	0.036	0.763	0.925
F1-score	U-Net [16]	0.564	0.095	0.027	0.503	0.624
	SegNet [15]	0.562	0.124	0.036	0.383	0.581
	FCN [17]	0.510	0.078	0.022	0.400	0.531
	Deeplabv3+ [18] with MobileNet [19]	0.640	0.241	0.069	0.487	0.794
	Deeplabv3+ [18] with ResNet [20]	0.480	0.262	0.075	0.313	0.646
	Deeplabv3+ [18] with Xception [21]	0.621	0.259	0.047	0.456	0.786
	Proposed method	**0.749**	0.188	0.054	0.629	0.868
mIoU	U-Net [16]	0.473	0.114	0.331	0.400	0.546
	SegNet [15]	0.380	0.129	0.037	0.298	0.462
	FCN [17]	0.363	0.086	0.025	0.308	0.418
	Deeplabv3+ [18] with MobileNet [19]	0.504	0.229	0.066	0.358	0.650
	Deeplabv3+ [18] with ResNet [20]	0.344	0.213	0.031	0.208	0.480
	Deeplabv3+ [18] with Xception [21]	0.487	0.251	0.072	0.327	0.647
	v3_DCNN-1280 * [22]	0.685	-	-	-	-
	Xception-65 * [23]	0.645	-	-	-	-

* The reported numbers of Guo [22] and Priego [23] are referred in this table.

**Table 2:** Multiple comparisons for segmentation of metastases on H–E WSIs: LSD test.

LSD Multiple Comparisons							
**Dependent Variable**	**(I) Method**	**(J) Method**	**Mean Difference (I–J)**	**Std. Error**	**Sig.**	**95**% **C.I.**	
						**Lower Bound**	**Upper Bound**
Precision	Proposed method	U-Net [16]	0.405 *	0.088	<0.001	0.311	0.499
		SegNet [15]	0.344 *	0.088	<0.001	0.250	0.438
		FCN [17]	0.339 *	0.088	<0.001	0.245	0.434
		Deeplabv3+ [18] with MobileNet [19]	0.248 *	0.088	<0.001	0.163	0.516
		Deeplabv3+ [18] with ResNet [20]	0.248 *	0.088	<0.001	0.072	0.424
		Deeplabv3+ [18] with Xception [21]	0.278 *	0.088	<0.001	0.102	0.454
Recall	Proposed method	U-Net [16]	0.195 *	0.079	<0.001	0.116	0.273
		SegNet [15]	0.249 *	0.079	<0.001	0.170	0.328
		FCN [17]	0.337 *	0.079	<0.001	0.258	0.416
		Deeplabv3+ [18] with MobileNet [19]	0.155 *	0.079	<0.001	0.350	0.313
		Deeplabv3+ [18] with ResNet [20]	0.397 *	0.079	<0.001	0.239	0.556
		Deeplabv3+ [18] with Xception [21]	0.253 *	0.079	<0.001	0.0.94	0.411
F1-score	Proposed method	U-Net [16]	0.280 *	0.075	<0.001	0.190	0.369
		SegNet [15]	0.382 *	0.075	<0.001	0.292	0.471
		FCN [17]	0.339 *	0.075	<0.001	0.304	0.482
		Deeplabv3+ [18] with MobileNet [19]	0.203 *	0.075	<0.001	0.524	0.354
		Deeplabv3+ [18] with ResNet [20]	0.364 *	0.075	<0.001	0.213	0.515
		Deeplabv3+ [18] with Xception [21]	0.222 *	0.075	<0.001	0.234	0.235
mIoU	Proposed method	U-Net [16]	0.275 *	0.055	<0.001	0.165	0.386
		SegNet [15]	0.369 *	0.055	<0.001	0.258	0.480
		FCN [17]	0.385 *	0.055	<0.001	0.275	0.496
		Deeplabv3+ [18] with MobileNet [19]	0.245 *	0.074	<0.001	0.096	0.393
		Deeplabv3+ [18] with ResNet [20]	0.405 *	0.074	<0.001	0.256	0.553
		Deeplabv3+ [18] with Xception [21]	0.261 *	0.074	<0.001	0.113	0.410

* The proposed method is significantly better than the baseline approaches using the LSD test (*p* < 0.001).

**Table 3:** Comparison of hardware and computing efficiency.

Method	CPU	RAM	GPU	Inference Time per WSI ${}^{\tau }$ (min.)
Proposed Method (with 4 GPUs)	Intel Xeon Gold 6134 CPU @ 3.20 GHz × 16	128 GB	4 × GeForce GTX 1080 Ti	**2.4**
Proposed Method (with 1 GPU)	Intel Xeon CPU E5-2650 v2 @ 2.60 GHz × 16	32 GB	1 × GeForce GTX 1080 Ti	**9.6**
U-Net [16]	Intel Xeon CPU E5-2650 v2 @ 2.60 GHz × 16	32 GB	1 × GeForce GTX 1080 Ti	44
SegNet [15]	Intel Xeon CPU E5-2650 v2 @ 2.60 GHz × 16	32 GB	1 × GeForce GTX 1080 Ti	43
FCN [17]	Intel Xeon CPU E5-2650 v2 @ 2.60 GHz × 16	32 GB	1 × GeForce GTX 1080 Ti	48
Deeplabv3+ [18] with MobileNet [19]	Intel Xeon CPU E5-2650 v2 @ 2.60 GHz × 16	32 GB	1 × GeForce GTX 1080 Ti	17.2
Deeplabv3+ [18] with ResNet [20]	Intel Xeon CPU E5-2650 v2 @ 2.60 GHz × 16	32 GB	1 × GeForce GTX 1080 Ti	18.2
Deeplabv3+ [18] with Xception [21]	Intel Xeon CPU E5-2650 v2 @ 2.60 GHz × 16	32 GB	1 × GeForce GTX 1080 Ti	17.8
v3_DCNN-1280 [22]	-	-	1 × GeForce GTX 1080 Ti	13.8 ${}^{\iota }$
Xception-65 [23]	Intel Xeon CPU E5-2698 v4 @ 2.2 GHz	256 GB	4 × Tesla V100 Tensor Core	398.2 *

τ The size of the WSI in this evaluation is 25,970,844,816 pixels (113,501 × 228,816 pixels). ι v3_DCNN [22] takes 11.5 min for a WSI with 97,792×221,184 pixels; 13.8 min=(11.5×(113,501×228,816))/(97,792×221,184). * The patch-based method [23] takes 0.23 s for a 500×500 patch; 398.2 min $=(0.23\;s\times \left\lfloor \frac{113,501}{500}\right\rfloor \times \left\lfloor \frac{228,816}{500}\right\rfloor )/60$ s.

## Data Availability

The data that support the findings of this study are available from the corresponding author upon reasonable request.
